# Correction: Stimulation of the left dorsolateral prefrontal cortex with slow rTMS enhances verbal memory formation

**DOI:** 10.1371/journal.pbio.3001451

**Published:** 2021-11-03

**Authors:** Mircea van der Plas, Verena Braun, Benjamin Johannes Stauch, Simon Hanslmayr

The email address for the corresponding author, Simon Hanslmayr, is incorrect. Dr. Hanslmayr’s email address is: simon.hanslmayr@glasgow.ac.uk
