# Supplementary material for: Stimulation of the left dorsolateral prefrontal cortex with slow rTMS enhances verbal memory formation
Source: PLoS Biol. 2021 Sep 28;19(9):e3001363. doi: 10.1371/journal.pbio.3001363 (PMC8478201; doi:10.1371/journal.pbio.3001363)
Supplement: S1 Fig — The plots contain the averaged activity from selected channels represented by red dots on the accompanying topography. Selected channels are characterised by a significant difference in beta power (i.e., less power in the DLPFC condition compared to the vertex condition). Word onset occurred at 0 s (indicated by dashed line). The data and scripts used to generate this figure can be found at https://osf.io/dyxjv/. DLPFC, dorsolateral prefrontal cortex. (DOCX) [file pbio.3001363.s001.docx]

**Supplementary Material S1 Fig: Individual time-frequency representations for list 2 items**


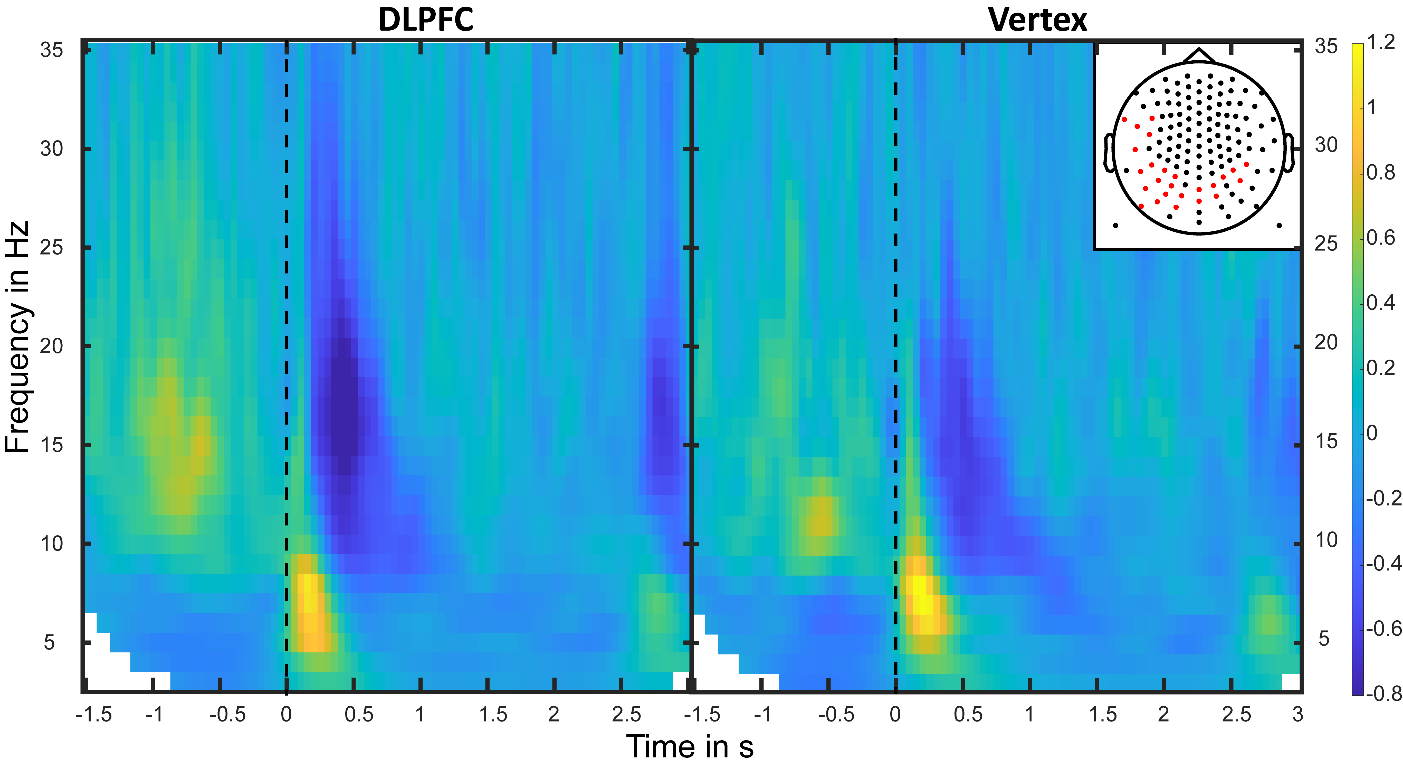
 *S1 Fig: Time Frequency Representations for list 2 trials during encoding for the DLPFC and vertex stimulation condition, respectively. The plots contain the averaged activity from selected channels represented by red dots on the accompanying topography. Selected channels are characterized by a significant difference in beta power (i.e. less power in the DLPFC condition compared to the vertex condition). Word onset occurred at 0 s (indicated by dashed line).*
