# Supplementary material for: Stimulation of the left dorsolateral prefrontal cortex with slow rTMS enhances verbal memory formation
Source: PLoS Biol. 2021 Sep 28;19(9):e3001363. doi: 10.1371/journal.pbio.3001363 (PMC8478201; doi:10.1371/journal.pbio.3001363)
Supplement: S2 Fig — Each raincloud plot is paired with its respective box plots. Coloured areas within the box plots indicate the standard error, while the circles depict individual data points for each participant, respectively. The same participants for the pre- and post-time windows are connected by a line. The thick line illustrates the change in mean from pre to post. (A) Raincloud plot of the alpha periodical component. (B) Raincloud plots of the beta periodical component. (C) Raincloud plots per stimulation condition for the offset of the aperiodical component at 0 Hz for pre- and poststimulus period word onset time windows, respectively. Yellow line represents an identical aperiodical component with an increased offset. (D) Raincloud plots per stimulation condition for the Exponent of the aperiodical component Hz poststimulus period windows, respectively. Yellow line represents an example for an identical component with a larger exponent. The data and scripts used to generate this figure can be found at https://osf.io/dyxjv/. DLPFC, dorsolateral prefrontal cortex. (DOCX) [file pbio.3001363.s002.docx]

*
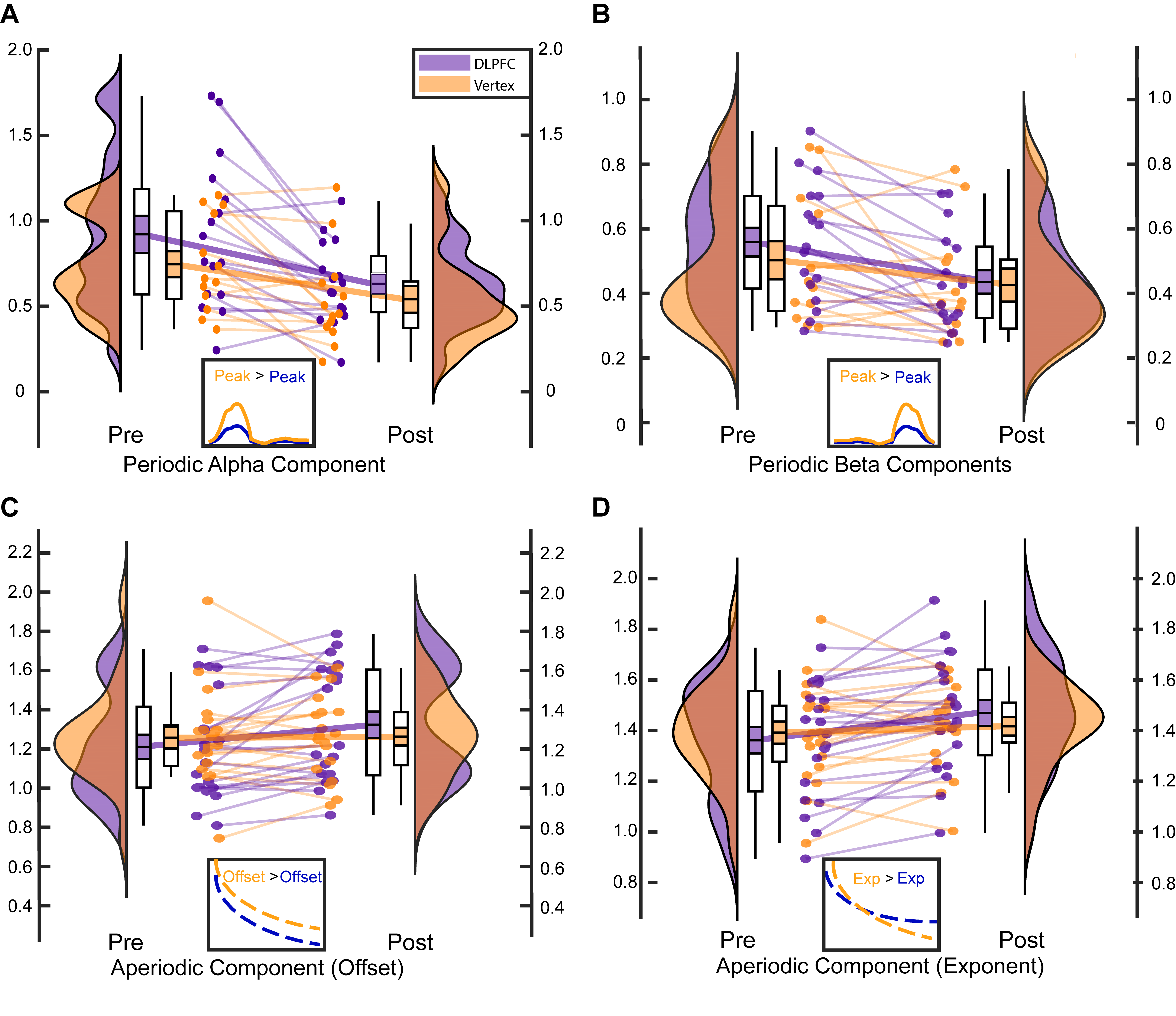
***Supplementary Material S2 Fig: Separated FOOOF Components per Condition and Frequency Band**

*S2 Fig: Raincloud plots of the components resulting from the FOOOF analysis for the DLPFC condition (purple) and the vertex condition (Orange), for the pre and the post stimulus period, respectively. Each raincloud plot is paired with its respective boxplots. Coloured area within the box-plots indicate the standard error, while the circles depict individual data points for each participant respectively. The same subjects for the pre and post time-windows are connected by a line. The thick line illustrates the change in mean from pre to post. A) Raincloud plot of the alpha periodical component; B) Raincloud plots of the beta periodical component; C) Raincloud plots per stimulation condition for the offset of the aperiodical component* *at 0 Hz for pre and post stimulus period, respectively word onset time windows. Yellow line represents an identical aperiodical component with an increased offset. D) Raincloud plots per stimulation condition for the Exponent of the aperiodical component Hz post stimulus period, respectivelywindows. Yellow line represents an example for an identical component with a larger exponent.*
