# Supplementary material for: Stimulation of the left dorsolateral prefrontal cortex with slow rTMS enhances verbal memory formation
Source: PLoS Biol. 2021 Sep 28;19(9):e3001363. doi: 10.1371/journal.pbio.3001363 (PMC8478201; doi:10.1371/journal.pbio.3001363)
Supplement: S3 Fig — (A) Raincloud plot of time difference between the first occurrence of a TMS pulse post-word presentation for every trial (N = 2,400; 1,200 per condition). Coloured areas within the box plots indicate the standard error, while the circles depict individual data points for each participant, respectively. A slight 4-Hz bias in timing is visible in both conditions based on how the ISI was implemented. With a perfectly random ISI, a uniform distribution would be expected. However, a 2-sample Kolmogorov–Smirnov test confirmed that these 2 distributions do not statistically differ from each other (k-s statistic: 0.0295; p = 0.6709). The data and scripts used to generate this figure can be found at https://osf.io/dyxjv/. DLPFC, dorsolateral prefrontal cortex; TMS, transcranial magnetic stimulation. (DOCX) [file pbio.3001363.s003.docx]

**Supplementary Material S3 Fig: TMS pulse shift Distribution**


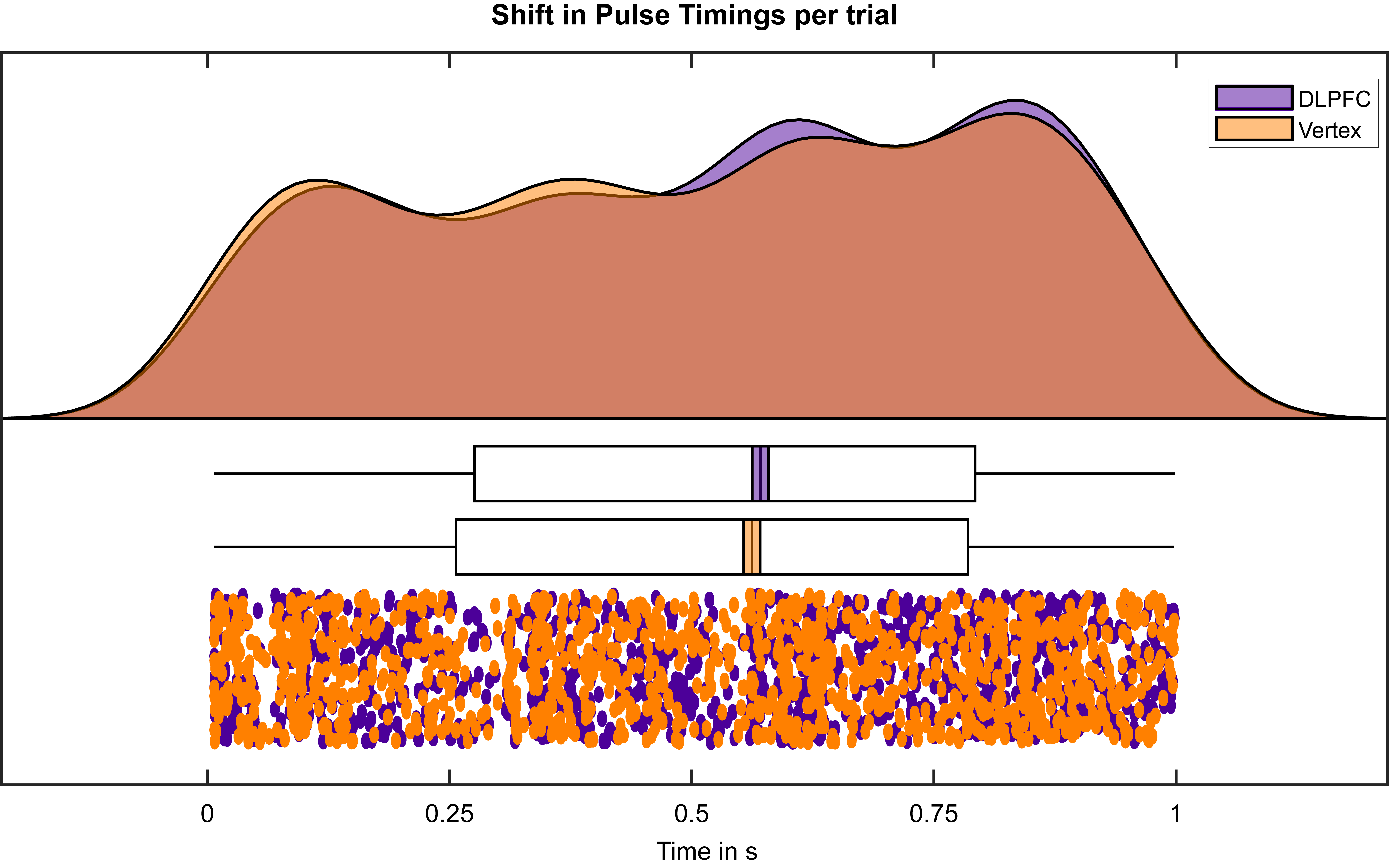


*S3 Fig: A) Raincloud plot of time difference between the first occurrence of a TMS pulse post word presentation for every trial (N=2400; 1200 per condition). Coloured area within the box-plots indicate the standard error, while the circles depict individual data points for each participant respectively. A slight 4 Hz bias in timing is visible in both conditions based on how the ISI was implemented. With a perfectly random ISI a uniform distribution would be expected. However, a two-sample Kolmogorov-Smirnov test confirmed that these two distributions do not statistically differ from each other* *(k-s statistic: 0.0295; p = 0.6709) .*
