# Supplementary material for: Stimulation of the left dorsolateral prefrontal cortex with slow rTMS enhances verbal memory formation
Source: PLoS Biol. 2021 Sep 28;19(9):e3001363. doi: 10.1371/journal.pbio.3001363 (PMC8478201; doi:10.1371/journal.pbio.3001363)
Supplement: S1 Text — (DLPFC: mean = 23.1, SD = 7.48; vertex: mean = 17.25, SD = 8.48). Power is not systematically biased by trial numbers, but we nevertheless tested whether this difference in trial numbers might have contributed to the observed effects. To this end, we randomly selected trials for each participant from the DLPFC group and matched these to the number of trials from participants in the vertex group, ensuring that both groups have exactly the same trial numbers (mean: 17.25, SD: 8.48). As our main comparison of interest was the difference in beta power (13–30 Hz) between the DLPFC and vertex group for List 2 trials, we conducted independent samples t tests for data 0–1 s after word onset averaged over the negative electrode cluster identified earlier. This procedure was repeated 100 times, every time randomly selecting new subsets of trials for the DLPFC group. Approximately 100 t tests on adjusted trial numbers revealed t values ranging from −3.9 to −2.377 (critical t for independent samples t tests = 2.023; df = 38). This analysis demonstrates that the difference in poststimulus beta power decreases for List 2 words was not driven by differences in trial numbers. DLPFC, dorsolateral prefrontal cortex. (DOCX) [file pbio.3001363.s004.docx]

**Supplementary Material S1 Text: Addressing Difference in Trial Numbers**

There was a considerable difference in the number of list 2 hits between the DLPFC and the vertex group because of the enhanced memory performance in the DLPFC group. (DLPFC: mean=23.1, SD=7.48; vertex: mean=17.25, SD=8.48). Power is not systematically biased by trial numbers, but we nevertheless tested whether this difference in trial numbers might have contributed to the observed effects. To this end, we randomly selected trials for each subject from the DLPFC group and matched these to the number of trials from subjects in the vertex group, ensuring that both groups have exactly the same trial numbers (mean: 17.25, SD: 8.48). As our main comparison of interest was the difference in beta power (13-30Hz) between the DLPFC and vertex group for list 2 trials, we conducted independent samples t-tests for data 0-1 s after word onset averaged over the negative electrode cluster identified earlier. This procedure was repeated 100 times, every time randomly selecting new subsets of trials for the DLPFC group. 100 t-tests on adjusted trial numbers revealed t values ranging from -3.9 to -2.377 (critical t for independent samples t-tests = 2.023; df=38). This analysis demonstrates that the difference in post-stimulus beta power decreases for list 2 words was not driven by differences in trial numbers.
