# Supplementary material for: Stimulation of the left dorsolateral prefrontal cortex with slow rTMS enhances verbal memory formation
Source: PLoS Biol. 2021 Sep 28;19(9):e3001363. doi: 10.1371/journal.pbio.3001363 (PMC8478201; doi:10.1371/journal.pbio.3001363)
Supplement: S1 Table — Since none of the factors showed a significant difference, the effects cannot be attributed due to differences in model fits. DLPFC, dorsolateral prefrontal cortex. (DOCX) [file pbio.3001363.s005.docx]

**Supplementary Material S1 Table: FOOOF model fits and error terms**

To ensure that any observed effects of the FOOOF analysis are legitimate and not a result due to differences in model fit we ran two 2 (within factor time: pre vs post) x 2 (between factor stimulation, DLPFC vs Vertex) mixed ANOVAs as control analyses. Since none of the factors showed a significant difference, the effects cannot be attributed due to differences in model fits:

## Mixed Anova of Model fit (R^2^)

| **Within Subjects Effects** | | | | | | | | | | | | | |
| --- | --- | --- | --- | --- | --- | --- | --- | --- | --- | --- | --- | --- | --- |
| **Cases** | | **Sum of Squares** | | **df** | | **Mean Square** | | **F** | | **p** | | **η²** | |
| Time |  | 0.002 |  | 1 |  | 0.002 |  | 3.706 |  | 0.062 |  | 0.053 |  |
| Time * Stimulation |  | 4.805e -5 |  | 1 |  | 4.805e -5 |  | 0.111 |  | 0.740 |  | 0.002 |  |
| Residuals |  | 0.016 |  | 38 |  | 4.310e -4 |  |  |  |  |  |  |  |
|  | | | | | | | | | | | | | |
| Note.  Type III Sum of Squares | | | | | | | | | | | | | |

| **Between Subjects Effects** | | | | | | | | | | | | | |
| --- | --- | --- | --- | --- | --- | --- | --- | --- | --- | --- | --- | --- | --- |
| **Cases** | | **Sum of Squares** | | **df** | | **Mean Square** | | **F** | | **p** | | **η²** | |
| Stimulation |  | 1.540e -4 |  | 1 |  | 1.540e -4 |  | 0.489 |  | 0.489 |  | 0.005 |  |
| Residuals |  | 0.012 |  | 38 |  | 3.149e -4 |  |  |  |  |  |  |  |
|  | | | | | | | | | | | | | |
| Note.  Type III Sum of Squares | | | | | | | | | | | | | |

## Mixed Anova of Residuals

| **Within Subjects Effects** | | | | | | | | | | | | | |
| --- | --- | --- | --- | --- | --- | --- | --- | --- | --- | --- | --- | --- | --- |
| **Cases** | | **Sum of Squares** | | **df** | | **Mean Square** | | **F** | | **p** | | **η²** | |
| Time |  | 0.002 |  | 1 |  | 0.002 |  | 3.037 |  | 0.089 |  | 0.026 |  |
| Time*stimulation |  | 9.138e -5 |  | 1 |  | 9.138e -5 |  | 0.145 |  | 0.706 |  | 0.001 |  |
| Residuals |  | 0.024 |  | 38 |  | 6.316e -4 |  |  |  |  |  |  |  |
|  | | | | | | | | | | | | | |
| Note.  Type III Sum of Squares | | | | | | | | | | | | | |

| **Between Subjects Effects** | | | | | | | | | | | | | |
| --- | --- | --- | --- | --- | --- | --- | --- | --- | --- | --- | --- | --- | --- |
| **Cases** | | **Sum of Squares** | | **df** | | **Mean Square** | | **F** | | **p** | | **η²** | |
| Stimulation |  | 9.668e -4 |  | 1 |  | 9.668e -4 |  | 0.787 |  | 0.381 |  | 0.013 |  |
| Residuals |  | 0.047 |  | 38 |  | 0.001 |  |  |  |  |  |  |  |
|  | | | | | | | | | | | | | |
| Note.  Type III Sum of Squares | | | | | | | | | | | | | |
