# Supplementary material for: Stimulation of the left dorsolateral prefrontal cortex with slow rTMS enhances verbal memory formation
Source: PLoS Biol. 2021 Sep 28;19(9):e3001363. doi: 10.1371/journal.pbio.3001363 (PMC8478201; doi:10.1371/journal.pbio.3001363)
Supplement: S2 Table — (DOCX) [file pbio.3001363.s006.docx]

**Supplementary Material S2 Table: Table containing temporal clustering scores per List**

| **List** | | **Stimulation** | | **Mean** | | **SD** | | **N** | |
| --- | --- | --- | --- | --- | --- | --- | --- | --- | --- |
| L1 |  | DLPFC |  | 4.163 |  | 1.697 |  | 18 |  |
|  |  | Vertex |  | 4.844 |  | 3.131 |  | 18 |  |
| L2 |  | DLPFC |  | 4.381 |  | 2.142 |  | 18 |  |
|  |  | Vertex |  | 4.484 |  | 2.261 |  | 18 |  |
|  | | | | | | | | | |

*S2 Table: Mean temporal clustering values per condition, with the accompanying standard deviation.*
