# Supplementary material for: Stimulation of the left dorsolateral prefrontal cortex with slow rTMS enhances verbal memory formation
Source: PLoS Biol. 2021 Sep 28;19(9):e3001363. doi: 10.1371/journal.pbio.3001363 (PMC8478201; doi:10.1371/journal.pbio.3001363)
Supplement: Attachment — Submitted filename: Response to Reviewers - Revision.docx [file pbio.3001363.s007.docx]

**Response to Reviewers:**

We wish to thank the reviewers for their careful reading of our manuscript and for providing such constructive feedback. In the following text we will address every point made by the reviewers. We provide the original points of the reviewers in Bold, our response to the comment in plain text, and the changed section in the manuscript in Italics. Within the manuscript, changes are indicated with comments and tracked changes (line references refer to the untracked version).

**Reviewer #1:**

1) **The major claim of the paper is that DLPFC stimulation affects encoding at the item level, potentially through feedback interactions with processing in posterior cortical regions. Behavioral evidence for this claim comes by comparing recall rates in Experiments 1 and 2, as well as measures of temporal organization in Experiment 2, to rule out an effect of stimulation on other memory processes. However, in the experimental paradigm recall of both list 1 and list 2 items occur at the same time. As a result, recall performance on the stimulated and non-stimulated lists are no longer independent from one another. This complicates the ability to interpret both the behavioral processes at play and the effect of stimulation. Given the delayed recall paradigm (and the resulting tendency to initiate recall at the beginning of list 1), recall of list 1 items can interfere with the ability to recall items on list 2. One potential mechanism by which stimulation may modulate recall
   performance is by reducing interference between the two lists. For example, DLPFC could elicit more similar contextual states across lists 1 and 2 in the DLPFC as compared to vertex stimulation condition. This contextual similarity would boost recall for list 2 in the DLPFC condition, while making it more difficult to access list 2 items in the vertex condition. This is just one of many potential behavioral explanations for the findings. In ruling out alternative explanations, I believe the authors only compared temporal clustering measures for list 2 between DLPFC and vertex conditions. To help interpret these results, it would be helpful to describe more accurately what was done, as well as report measures on each list (e.g., what were temporal clustering scores for each condition and list). In addition, it would be useful to compare clustering by list (i.e., did subjects cluster items from list 1 or list 2 together in their recall sequences) in each stimulation condition
   to rule out other potential contextual effects. In general, additional forms of evidence are necessary to make the claim that the effects are at a "word-specific" level.**

The reviewer makes a good point, but before addressing it we would like to clarify that the item specificity of DLPFC stimulation is not the major claim of the paper. Instead, the major claim of our paper is that DLPFC stimulation improves memory encoding. In the paper we suggest that this effect operates at an item level because temporal clustering did not seem to have been affected by the DLPFC stimulation.

The reviewer raises an interesting hypothesis in that DLPFC stimulation might have increased contextual similarity between list 1 and list 2. However, we would not have expected such an effect because DLPFC is arguably involved in temporal clustering of items given its role in working memory processes (Blumenfeld & Ranganath, 2006,  *J Neurosci; https://www.jneurosci.org/content/26/3/916.short*). Therefore, inhibiting the DLPFC with 1 Hz rTMS should disrupt this process and consequently reduce contextual binding.

To address the reviewer’s concern we have taken two steps.

1. We included a section on the temporal clustering analysis in the ‘Methods’ section to make the procedure more transparent to the reader.

*(line 535) Temporal Clustering*

*To investigate whether the observed memory effects could be explained due to contextual effects resulting from the stimulation, we calculated temporal clustering scores per participant for each respective list and stimulation condition (procedure is based on [61]).* *This procedure can be summarized with the formula:*

$$\sum_{n=1}^{R} |{ObservedDistance}_{(n)}-ExpectedDistance|$$

*Where the observed distance was defined as the absolute difference between the observed recall position and the position during encoding for each subsequently recalled item (R). For example, if a subject recalls an item in the 3^rd^ position and subsequently recalls an item in the 5^th^ position the observed distance would be 2. The expected difference is the distance value that would be expected during optimal temporal clustering (Expected difference = 1;* *e.g., one would expect the 4^th^ item to be recalled following the 3^rd^ item* *yielding a difference of 1).*

*This yielded a temporal clustering value for each condition for every participant (see supplementary material S6 for values per condition). These were then directly compared to each other using a 2x2 repeated measure ANOVA (List x Stimulation Condition).*

Lastly, we added a table containing the descriptive statistics of all the temporal clustering values in the supplementary material.

| **Supplementary Material S6: Table containing temporal clustering scores per List** | | | | | | | | | |
| --- | --- | --- | --- | --- | --- | --- | --- | --- | --- |
| **List** | | **Stimulation** | | **Mean** | | **SD** | | **N** | |
| L1 |  | DLPFC |  | 4.163 |  | 1.697 |  | 18 |  |
|  |  | Vertex |  | 4.844 |  | 3.131 |  | 18 |  |
| L2 |  | DLPFC |  | 4.381 |  | 2.142 |  | 18 |  |
|  |  | Vertex |  | 4.484 |  | 2.261 |  | 18 |  |
|  | | | | | | | | | |

*S6: Mean temporal clustering values per condition, with the accompanying standard deviation.*

2. As requested by the reviewer we have updated the analysis to compare the contextual error/temporal clustering values for List 1 and List 2 items for both stimulation conditions. Thus, unlike the original t-test, the analysis has been extended to a repeated measures ANOVA with both List and Stimulation condition as a factor (since these are both within subject variables in Experiment 2). This analysis also does not indicate any differences for temporal clustering across conditions as evident by a non-significant List by Stimulation interaction.

*(line 219) For most of the participants (N=18 ), the order in which words were recalled was also available. This allowed us to assess the amount of temporal clustering [25] for list 1 and list 2 words and to examine whether DLPFC stimulation affected the amount of contextual error. Such an effect would be predicted by theories implicating the DLPFC in organizing memory material into temporal clusters [26]. A 2 (List 1 vs List 2) x 2 (DLPFC vs vertex) repeated measures ANOVA was conducted to determine whether temporal clustering is affected by stimulation. No significant main effects or interaction were observed (main effect for Stimulation: F(1,17)=0.624, p=0.440, η2p=0.012; main effect for List: F(1,17)=0.017, p=0.899, η2p=0.003; interaction List x Stimulation: F(1,17)=0.452, p=0.511, η2p=0.007).* *To ensure that we did not miss a potential effect of temporal clustering for list 2 items between the stimulation conditions, we performed a post hoc follow-up t-test on the List 2 items only, which also failed to show a significant difference between stimulation conditions (List 2 DLPFC vs List 2 Vertex: t(1,17)=-0.109, 0.914). These results indicate that the memory enhancement effect of left DLPFC stimulation cannot be attributed to changes in temporal clustering of the words between or within lists. Rather, DLPFC stimulation seemed to have improved memory performance for each item independently.*

2) **The stimulus pool consists of 240 nouns from the MRC psycholinguistic database. From my reading of the methods, it does not appear semantic similarity was controlled for on a list-by-list basis or between stimulation and non-stimulation lists for a single recall trial. Is it possible that any of the observed effects were related to semantic organization of recall?**

We appreciate the concern of the reviewer, but we think that this issue has been addressed by the design of the experiment. While semantic similarity was not explicitly tested (as in: Manning, & Kahana, 2012, *Memory,* *https://doi.org/10.1080/09658211.2012.683010*), lists were counterbalanced across conditions (List 1, List 2, DLPFC, and Vertex) in both experiments (as described in the methods section under ‘Task and Stimulation Material’ sections for the two experiments). The word material was initially split into 24 lists, each containing 10 words. These lists were then assigned to 6 blocks, reflecting the six repetitions per condition. Each block therefore contained 4 word lists, which were labelled A, B, C, and D. The assignment of these four lists to conditions (Forget-List1, Forget-List2, Remember-List1, Remember List2) was counterbalanced across participants, such that each list occurred equally often in each condition. For experiment 1 (between subjects design), subjects in the DLPFC stimulation condition and the Vertex stimulation condition received the exact same lists. In the second experiment (within subjects design), every list occurred equally often in each condition across subjects. Consequently, if for instance one list (say list A) was incidentally more semantically organized than the other lists then it would have affected both stimulation conditions equally. Therefore, we do not think that there were any confounds between stimulation condition and semantic similarity of the word material. Any systematic effects (such as DLPFC stimulation), in our opinion, cannot therefore not be explained by material effects such as semantic organization.

3) **I am not convinced that the reported EEG results, including the analysis of model parameters from the FOOOF toolbox, provide an accurate description of how stimulation affected neurophysiology. The authors are well-motivated in their analysis of the beta band (13-30 Hz) from prior work. However, from inspection of the power spectra reported in Figure 4b, there appear to be sideband effects from alpha oscillations that bleed into this frequency range. The reported effect of stimulation on memory may in fact be explained by this effect. Are effects in this beta band correlated with effects in the alpha range? Are there oscillatory effects of stimulation at alpha (i.e., does the amplitude of alpha oscillations from the FOOOF analysis differ between these conditions)?**

We appreciate that the reviewer suspects that some of the reported EEG results might be due to a potential effect in the alpha band based on the raw power-spectra. Indeed, we had a similar thought when we first saw these spectra. However, it turns out that the alpha changes are not systematically linked to the stimulation itself and therefore disappear after normalizing the spectra (as is visible in the difference in the z-scored time-frequency plots contrasting the two stimulation conditions in figure 4b). Nevertheless, and to be sure that we did not miss any effects in alpha, we also conducted a FOOOF analysis considering peaks in the alpha band (8-15 Hz). This analysis returned no significant interaction effect between the stimulation condition and timing (pre v post) on the periodic component of alpha (PREPOST x STIMULATION: F(1,32)=0.612 p=0.440, η^2^_p_=0.019).

We integrated this addition to the analysis in the results section in the following way:

*(line 157) Recent research suggests that some broadband memory-related effects are driven by a change in spectral tilt (i.e. aperiodic components) rather than a change in narrow band oscillations (i.e. periodic components) [22]. To investigate if the above reported effect of DLPFC stimulation on beta power is due to a change in oscillatory activity or a change in spectral tilt, we separated power-spectra into periodic and aperiodic components using the FOOOF toolbox (see figure 4A for schematic representation of the components as labelled by FOOOF) [23]. Moreover, we included components in the alpha band in this analysis, as the raw power spectra exhibited prominent alpha peaks (see figure 4B).*

*We performed a 2 (pre vs post: TIME) x 2 (DLPFC vs vertex: STIMULATION) repeated-measurements ANOVA on the periodic and aperiodic components respectively, with TIME as a within subjects factor and STIMULATION as a between subjects factor. We observed a significant interaction effect for the aperiodic component, as reflected by the exponent and offset of the aperiodic component: Exponent: PREPOST x STIMULATION: F(1,38)=5.900, p=0.020, η2p=0.134; Offset: PREPOST x STIMULATION F(1,38)=5.646, p=0.023, η2p=0.129 (see Figure 4 , for the distributions of the separate components see supplementary material S2). No such interaction effect was observed for the ANOVA investigating the periodic/oscillatory activity in the beta frequency band (PREPOST x STIMULATION: F(1,27)=0.652 p=0.426, η2p=0.024) or alpha frequency band (PREPOST x STIMULATION: F(1,32)=0.612 p=0.440, η2p=0.019). For both these components only a TIME effect could be observed (beta: TIME: F(1,27)=012.267 p=0.002, η2p=0.312) alpha: TIME: F(1,32)=26.471 p=0.001, η2p=0.453). These results suggest that the interaction observed in the time-frequency representation was mainly driven by the aperiodic component, rather than narrow band oscillatory beta or alpha activity. In particular, the results suggest that DLPFC stimulation leads to a steeper aperiodic component where power decreases more quickly as frequency increases.*

We also adjusted the supplementary figure showcasing the effects in more details to include the alpha frequency analysis as well:

*S2: Raincloud plots of the components resulting from the FOOOF analysis. for the DLPFC condition (purple) and the vertex condition (Orange), for the pre and the post stimulus period, respectively.* *Each raincloud plot is paired with its respective boxplots. Coloured area within the box-plots indicate the standard error, while the circles depict individual data points for each participant, respectively. The same subjects for the pre and post time-windows are connected by a line. The thick line illustrates the change in mean from pre to post. A) Raincloud plot of the alpha periodical component; B) Raincloud plots of the beta periodical component; C) Raincloud plots per stimulation condition for the offset of the aperiodical component at 0 Hz for pre and post stimulus period, respectively . Yellow line represents an identical aperiodical component with an increased offset. D) Raincloud plots per stimulation condition for the Exponent of the aperiodical component at 0 Hz for pre and post stimulus period, respectively word onset time windows . Yellow line represents an example for an identical component with a larger exponent.*

4) **The authors chose to make inferences on the power spectra based off parameters in the 1/f fit, finding a potentially interesting effect in the slope in log-log space. However, it is difficult to interpret these model parameters for several reasons. First, how well were the power spectra fit by the model? Differences between conditions could in fact be driven by issues with model fit rather than actual changes in the power spectra. A comparison of residuals between experimental conditions, broken down by frequency, would alleviate this concern. Second, is it possible to distinguish whether the effects of stimulation are during stimulus processing itself vs. the pre-stimulus period? As TMS was randomly timed relative to stimulus presentation, it would be useful to know whether the effects are related to stimulus processing, as interpreted by the authors.**

The reviewer brings up an important point regarding the FOOOF analysis. To tackle this issue, we contrasted the model fits (r^2^) and the residuals between conditions. We performed two mixed 2 (within factor time: pre, post) x 2 (between factor stimulation: DLPFC, Vertex) ANOVAs and added the results in the supplementary materials and refer to it in the methods section. No significant main effects or interactions were observed for the model fit or the residuals. We included the results of this analysis as a table in the supplementary material section S5, allowing the reader to see the complete results of this analysis and refer to this control analysis in the EEG Analysis section within the methods to bring the attention of the reader to this analysis.

*(line 496)* *Additionally a control analysis was performed to ensure that there was no difference in model fits and residuals between the different FOOOF models that could alternatively explain any of the effects presented in this study (see supplementary material S5)*

***Supplementary Material S5: FOOOF model fits and error terms***

*To ensure that any observed effects of the FOOOF analysis are legitimate and not a result due to differences in model fit we ran two 2 (within factor time: pre vs post) x 2 (between factor stimulation, DLPFC vs Vertex) mixed ANOVAs as control analyses. Since none of the factors showed a significant difference, the effects cannot be attributed due to differences in model fits:*

## *Mixed Anova of Model fit (R^2^)*

| ***Within Subjects Effects*** | | | | | | | | | | | | | |
| --- | --- | --- | --- | --- | --- | --- | --- | --- | --- | --- | --- | --- | --- |
| ***Cases*** | | ***Sum of Squares*** | | ***df*** | | ***Mean Square*** | | ***F*** | | ***p*** | | ***η²*** | |
| *Time* |  | *0.002* |  | *1* |  | *0.002* |  | *3.706* |  | *0.062* |  | *0.053* |  |
| *Time * Stimulation* |  | *4.805e -5* |  | *1* |  | *4.805e -5* |  | *0.111* |  | *0.740* |  | *0.002* |  |
| *Residuals* |  | *0.016* |  | *38* |  | *4.310e -4* |  |  |  |  |  |  |  |
|  | | | | | | | | | | | | | |
| Note.  *Type III Sum of Squares* | | | | | | | | | | | | | |

| ***Between Subjects Effects*** | | | | | | | | | | | | | |
| --- | --- | --- | --- | --- | --- | --- | --- | --- | --- | --- | --- | --- | --- |
| ***Cases*** | | ***Sum of Squares*** | | ***df*** | | ***Mean Square*** | | ***F*** | | ***p*** | | ***η²*** | |
| *Stimulation* |  | *1.540e -4* |  | *1* |  | *1.540e -4* |  | *0.489* |  | *0.489* |  | *0.005* |  |
| *Residuals* |  | *0.012* |  | *38* |  | *3.149e -4* |  |  |  |  |  |  |  |
|  | | | | | | | | | | | | | |
| Note.  *Type III Sum of Squares* | | | | | | | | | | | | | |

## *Mixed Anova of Residuals*

| ***Within Subjects Effects*** | | | | | | | | | | | | | |
| --- | --- | --- | --- | --- | --- | --- | --- | --- | --- | --- | --- | --- | --- |
| ***Cases*** | | ***Sum of Squares*** | | ***df*** | | ***Mean Square*** | | ***F*** | | ***p*** | | ***η²*** | |
| *Time* |  | *0.002* |  | *1* |  | *0.002* |  | *3.037* |  | *0.089* |  | *0.026* |  |
| *Time*stimulation* |  | *9.138e -5* |  | *1* |  | *9.138e -5* |  | *0.145* |  | *0.706* |  | *0.001* |  |
| *Residuals* |  | *0.024* |  | *38* |  | *6.316e -4* |  |  |  |  |  |  |  |
|  | | | | | | | | | | | | | |
| Note.  *Type III Sum of Squares* | | | | | | | | | | | | | |

| ***Between Subjects Effects*** | | | | | | | | | | | | | |
| --- | --- | --- | --- | --- | --- | --- | --- | --- | --- | --- | --- | --- | --- |
| ***Cases*** | | ***Sum of Squares*** | | ***df*** | | ***Mean Square*** | | ***F*** | | ***p*** | | ***η²*** | |
| *Stimulation* |  | *9.668e -4* |  | *1* |  | *9.668e -4* |  | *0.787* |  | *0.381* |  | *0.013* |  |
| *Residuals* |  | *0.047* |  | *38* |  | *0.001* |  |  |  |  |  |  |  |
|  | | | | | | | | | | | | | |
| Note.  *Type III Sum of Squares* | | | | | | | | | | | | | |

Regarding the second question, we think that the binned time-course analysis contrasting the EEG response for List 1 and List 2 trials (see paragraph starting at line 132) gives us insight into this question (see figure 3D). The List 2 - List 1 contrast for DLPFC condition trials showed that there was a significant difference both before (increased power for stimulation trials) and after stimulus (i.e. word) onset (decreased power in stimulation trials) compared to the Vertex condition, which acts as the control in this regard. Based on this result we think that DLPFC stimulation affects processing in both the pre-stimulus (by increasing beta power) and the post-stimulus period (by decreasing beta power). Previous literature suggests that such an increase in prestimulus power together with a pronounced decrease in poststimulus power is a particularly good scenario for memory performance (Klimesch, Doppelmayr, Hanslmayr, 2006; Prog Brain Res; <https://pubmed.ncbi.nlm.nih.gov/17071229/> )

5) **For the EEG analysis, the authors use a cluster-based permutation test to identify sensors that show differences in beta power between the DLPFC and vertex groups. Given the between-subject design of the experiment, and the claim that decreases in beta power reflects word-specific processing, would the relevant neural measure not be differences in beta power for subsequently recalled and not recalled items? If beta desynchronization is similar for subsequently recalled and forgotten items in the DLPFC condition or there are no differences in subsequent memory effects across stimulation conditions, this would have strong implications for the mechanism by which stimulation affected memory.**

We agree that a comparison between beta power for remembered and forgotten items would be interesting. However, this comparison can only be done in a meaningful way if enough trials are available for later remembered and later not remembered items. Unfortunately, due to the high performance of the subjects, especially in the DLPFC condition, this was not the case. A previous study by the senior author (Hanslmayr, Spitzer, and Bäuml, 2009, Cerebral Cortex) demonstrated that >13 trials per condition are needed to obtain stable estimates for alpha/beta power decreases. Only 6 subjects were available in the current dataset that would have fulfilled this criterion which is not enough for a meaningful analysis for subsequent memory effects.

Importantly, we do not think that leaving this comparison out detracts from the core finding of this paper. The main finding of this paper is that DLPFC stimulation during memory encoding can boost memory performance in a list learning task. A good way to show that a specific stimulation paradigm leads to behavioural changes, is by showing that stimulation in a task-unrelated area does not produce similar memory enhancements as the task-relevant stimulation for recalled items only. If one were to compare remembered and forgotten items one would run the risk of introducing a bias resulting from trial imbalances (i.e. higher proportion of forgotten trials in the vertex compared to the DLPFC condition). This could introduce spurious effects when comparing trials between the DLPFC and vertex condition. Therefore, the fact that we do find more pronounced power decreases (for remembered trials only) for the DLPFC condition compared to the vertex condition is remarkable and evidence that the DLPFC stimulation did affect EEG power in a way that is favourable for memory. Moreover, comparing activity between remembered and forgotten items exclusively for the DLPFC condition would in our view be not much more informative as we would not be able to disentangle whether the observed power changes are due to stimulation or simply the result of a standard subsequent memory effect (for a review see Hanslmayr, & Staudigl, 2014, Neuroimage https://doi.org/10.1016/j.neuroimage.2013.05.121).

6) **Regarding scholarship, the idea that 1 Hz rTMS is generally inhibitory has recently been challenged (see Castrillon et al., 2020, Science Advances), as the effects depend on the network structure of the targeted region. Notably, stimulation of frontal sites with network properties similar to the DLPFC location in the current study decreased local inhibition and disrupted network connectivity. Discussion of whether the present findings support inhibition as the mechanism by which 1 Hz stimulation affects brain function may be warranted.**

We thank the reviewer for bringing this interesting publication to our attention. Indeed, the paper suggests, that 1 Hz offline TMS might not have a canonical effect throughout the whole cortex. While this is a very interesting finding it is not very easy to interpret their results, since the relationship between BOLD responses and excitation/inhibition is ambiguous (Logothetis & Wandell, 2004, *Annual Review of Physiology, https://www.annualreviews.org/doi/abs/10.1146/annurev.physiol.66.082602.092845*). The BOLD signal primarily reflects synaptic potentials, as opposed to neural spikes. Because both excitation and inhibition result in synaptic potentials the BOLD signal will reflect both. Indeed, the fact that Castrillon et al. did not observe any local changes directly under the coil, underlines this issue. Moreover, we mainly base the interpretation of 1Hz stimulation on the observation in intracranial work in mice showing that such paradigms lead to synaptic long-term depression (Dudek, & Bear, 1992, *Proc Natl Acad Sci U S A.*), which alongside long term potentiation (LTP) is often thought to be relevant for memory processes (Nakazawa, McHugh,Wilson, & Tonegawa, 2004, *Nat. Rev., Neurosci.).* In contrast to this online rTMS interpretation, Castrillon et al. instead focus on the effects of offline rTMS. However, we agree that the work by Castrillon supports a possible alternative explanation we put forward in the discussion, regarding the possible effects of rTMS on network activity as opposed to local inhibition per se. Therefore, we made sure to include it in the appropriate section.

*(line 317) Another possible interpretation, that disregards possible facilitative or inhibitory effects of rTMS, is that given our remote effects during left DLPFC stimulation, 1Hz rTMS may have influenced the functional connectivity between frontal and posterior regions [45]. This enhanced connectivity would then lead to enhanced stimulus processing and improved memory performance as a result thereof. Indeed, a recent study has shown that 1 Hz rTMS can have opposite effects on different networks [46]. Castrillon et al. found that while occipital stimulation led to signal propagation to down-stream areas, frontal stimulation disrupted network communication. Therefore, extrapolating this finding to the results presented in this paper, it is possible that the parietal beta power decrease is the result of a disrupted network communication, as opposed to local inhibition in the DLPFC per se.*

7) **The authors seem to imply that the effects of stimulation were specific to beta, as they state no effects were observed for alpha or theta frequencies. However, I assume this is because the authors filtered their analysis to sensors that showed a beta difference in the 1 second post item onset. If the authors use the same selection procedure for alpha or theta bands, are there no observed effects?**

The filtering in the beta band only occurred after a broad-band analysis (2:35 Hz) across all sensors had already been performed. Any further analyses on the sensors (such as the FOOOF analysis) merely serve to characterize the effect further. If there were any significant alpha or theta effects in any sensors they should have been discovered in the cluster permutation test. However, to ensure that we did not miss any effects we made sure to extend the FOOOF analysis to a wider variety of frequency bands of interest, including the theta (4-8 Hz) and alpha (8-12 Hz) bands. Regarding the theta band, there were only 2 subjects with distinct peaks in the theta band. This makes further analyses uninformative. Regarding the alpha band, there was no significant interaction effect between the stimulation condition and timing (pre v post) on the periodic component (PREPOST x STIMULATION: F(1,32)=0.612 p=0.440, η^2^_p_=0.019), just as in the beta band analysis. As previously stated in the response to comment 3 we integrated this additional analysis into the manuscript in the following ways:

*(line 157) Recent research suggests that some broadband memory-related effects are driven by a shift in spectral tilt (i.e. aperiodic components) rather than a change in narrow band oscillations (i.e. periodic components) [22]. To investigate if the above reported effect of DLPFC stimulation on beta power is due to a change in oscillatory activity or a change in spectral tilt, we separated power-spectra into periodic and aperiodic components using the FOOOF toolbox (see figure 4A for schematic representation of the components as labelled by FOOOF) [23]. Moreover, we included components in the alpha band in this analysis, as the raw power spectra exhibited prominent alpha peaks (see figure 4B).*

*We performed a 2 (pre vs post: TIME) x 2 (DLPFC vs vertex: STIMULATION) repeated-measurements ANOVA on the periodic and aperiodic components respectively, with TIME as a within subjects factor and STIMULATION as a between subjects factor. We observed a significant interaction effect for the aperiodic component, as reflected by the exponent and offset of the aperiodic component: Exponent: PREPOST x STIMULATION: F(1,38)=5.900, p=0.020, η2p=0.134; Offset: PREPOST x STIMULATION F(1,38)=5.646, p=0.023, η2p=0.129 (see Figure 4 , for the distributions of the separate components see supplementary material S2). No such interaction effect was observed for the ANOVA investigating the periodic/oscillatory activity in the beta frequency band (PREPOST x STIMULATION: F(1,27)=0.652 p=0.426, η2p=0.024) or alpha frequency band (PREPOST x STIMULATION: F(1,32)=0.612 p=0.440, η2p=0.019). For both these components only a TIME effect could be observed (beta: TIME: F(1,27)=012.267 p=0.002, η2p=0.312) alpha: TIME: F(1,32)=26.471 p=0.001, η2p=0.453). These results suggest that the interaction observed in the time-frequency representation was mainly driven by the aperiodic component, rather than narrow band oscillatory beta or alpha activity. In particular, the results suggest that DLPFC stimulation leads to a steeper aperiodic component where power decreases more quickly as frequency increases.*

We also adjusted the supplementary figure showcasing the effects in more details to include the alpha frequency analysis as well:

*S2: Raincloud plots of the components resulting from the FOOOF analysis. for the DLPFC condition (purple) and the vertex condition (Orange), for the pre and the post stimulus period, respectively.* *Each raincloud plot is paired with its respective boxplots. Coloured area within the box-plots indicate the standard error, while the circles depict individual data points for each participant, respectively. The same subjects for the pre and post time-windows are connected by a line. The thick line illustrates the change in mean from pre to post. A) Raincloud plot of the alpha periodical component; B) Raincloud plots of the beta periodical component; C) Raincloud plots per stimulation condition for the offset of the aperiodical component at 0 Hz for pre and post stimulus period, respectively . Yellow line represents an identical aperiodical component with an increased offset. D) Raincloud plots per stimulation condition for the Exponent of the aperiodical component at 0 Hz for pre and post stimulus period, respectively word onset time windows . Yellow line represents an example for an identical component with a larger exponent.*

**Minor issues:**

1) **It would be helpful to clarify the prediction justifying a one-tailed test for DLPFC vs. vertex effects in the methods.**

We thank the reviewer for pointing this out. We expanded the respective passage in the EEG-methods section.

*(line 472)* *Since we analysed the data in the context of an increased memory performance, which according to the sync/desync hypothesis should be characterized by cortical alpha/beta power decreases, only negative clusters were expected [3,14]. Therefore, data from the DLPFC and vertex group were subjected to a one-tailed cluster based permutation test, averaged over beta (13-30 Hz) and the post stimulus time window of interest (0 to 1 s).*

2) **It is unclear if the FOOOF analysis included frequencies above 40 Hz or below 2 Hz during model fitting. The methods claim 2-35 Hz, but the figures do not match this description. If these frequencies were excluded, they should be excluded from Figures 4B-D as the model was not fit to these frequencies.**

We apologize for the confusion caused by an omission on our part. The FOOOF analysis and TFR analysis were performed on differently processed signals. The 2-35 Hz only applies to the TFR analysis. For the FOOOF analysis we used bandpass filtered data in the 1-80 Hz frequency range allowing us to more optimally select the parameters that lead to the best fit and reducing the possibility of spurious effects resulting from the filters, for the frequencies of interest. The model was then subsequently fit to a 1-40 Hz frequency window of interest, as this provided the best fit results for the frequency bands of interest. The frequency representations exhibited a clear ‘knee’ in the power spectra past ~40 Hz, which was detrimental to the fits in the lower-frequencies. We made sure to specify this explicitly in the FOOOF methods.

*(line 486) The properties of observed power-changes were further investigated using the FOOOF toolbox [23]. This method uses simultaneous fitting of the aperiodic spectrum component as well as spectral peaks. For this we analysed a 1-80 Hz band-pass filtered signal in the time-window of interest (resulting from the time frequency analysis) and an identically sized time-window before stimulus presentation. This time-window was chosen to minimize any effects the filtering process might have on the frequency spectrum. The model was subsequently fit using a frequency range of interest of 1-40 Hz to optimize fits for the low-frequency (alpha and beta) bands of interest. These components were then be analysed separately. We performed a 2 x 2 mixed repeated measure ANOVA (Pre vs Post (2) word presentation x DLPFC vs vertex (2) stimulation, for each component (the aperiodic exponent, the offset and the periodic peak power). Additionally, a control analysis was performed to ensure that there were no differences in model fits or residuals between the different FOOOF models that could alternatively explain any of the effects presented in this study (see supplementary material S5)*

Regarding figure 4B, we think that it is reasonable to retain the x-axis as is, since this data was filtered in the same frequency range as the data used as input in the FOOOF analysis. The x-axes in figure 4C-D serve an illustrative purpose. The exponent and offset are a single value given by the slope of the line in that figure. Technically one could plot the lines to infinity. By extending the x-axis the difference in slopes as determined by the analysis become more visible to the reader. To highlight that the x-axis is extended for visual purposes we made sure to highlight this in the caption.

*(line 185) C)-D) Line plots of the mean aperiodic component before and after item presentation for the DLPFC and vertex condition, respectively. The right axis relates to the plotted post-pre difference (dotted line). The x-axis has been extended for illustrative purposes, to highlight the differences in slopes between the difference conditions. The actual fit was performed on data in the 1-40 Hz range.*

3) **It is unclear what the R graphic denotes when reading Figure 1. It would be helpful for the reader if this were explained in the caption.**

The R was supposed to represent the cue indicating that the list would have to be remembered. Due to this comment, and the comments made by the editor we have improved Figure 1 to provide more information about the experimental procedure.

4) **How were EEG data z-scored (e.g., across trials or time, within condition or across conditions, across all items or only recalled items)? The translation from the relatively raw data in Figure 4b to the time-frequency plot in Figure 3a are surprising, given the lack of any alpha signals in the latter.**

The EEG data was z-scored within the trial across time, that is within subject and within condition: for each trial and frequency bin we calculated the average power of the trial across time and subtracted it from the power at every given time-point. Then this difference was divided by the respective standard deviation, resulting in a z-score for each time point and frequency bin. We clarified this in the respective section within the manuscript:

*(line 469) EEG data (-1.5s to 3 s) were subjected to a time-frequency decomposition (2 to 35 Hz in steps of 1 Hz) using Morlet wavelets (width 7) and z-transformed* *per trial across time for each subject, within each stimulation condition, to enable analysis of post- as well as pre-stimulus activity [61]. The z-transformation was calculated by subtracting the average trial power across time and subtracting it from the power at every given timepoint. The difference was then divided by the respective standard deviation.*

Regarding the lack of alpha effects, it is important to mention that Figure 3a shows a contrast of DLPFC – Vertex, meaning that the alpha peaks were not affected by stimulation, and therefore probably subtracted out. To illustrate this point, we added the time frequency representations (TFRs) of the two respective stimulation conditions. One can appreciate the presence of increased prestimulus and decreased poststimulus alpha power which, however, does not differ between conditions. In contrast, these plots show a larger increase in beta power prestimlus and a larger decrease in beta power poststimulus in the DLPFC condition.

*S1: Time Frequency Representations for list 2 trials during encoding for the DLPFC and vertex stimulation condition, respectively. The plots contain the averaged activity from selected channels represented by red dots on the accompanying topography. Selected channels are characterized by a significant difference in beta power (i.e. less power in the DLPFC condition compared to the vertex condition). Word onset occurred at 0 s (indicated by dashed line).*

This material was integrated in the main text by referring to it in the following way:

*(line 126) The time frequency plot at this negative electrode cluster, as well as the time course of beta power, is shown in Figure 3A and 3C (for the individual time frequency plots for the DLPFC and vertex condition see supplementary material S1 ).*

Reviewer #2: Simon Davis

1) **Though it is acknowledged throughout the Intro/Disc/Methods that the original experiment was a directed forgetting paradigm, it would be useful to mention when the paradigm is first explained (e.g. near ln77) that the data analyzed here represents ½ of the previous data set, i.e. the runs without the "Forget" cues.**

We agree that it is important information and made sure to make this issue more explicit than in the previous draft, by reminding the reader that the analysed data only includes half of the originally recorded data at the beginning of the results section and explicitly highlighting this fact in figure 1.

*Experiment 1: Behaviour*

*(line 76) Participants were presented with two lists of ten words per encoding-retrieval run over the course of twelve runs. Following the six analysed lists, they were instructed to remember (i.e. keep in mind) the list just presented. After undertaking a short distractor task, participants were asked to recall all words from the two word lists just presented. The experimental group received 1 Hz rTMS to the left DLPFC during encoding of the second list and the control group received stimulation to the vertex (see Figure 1). It is important to note here, that the material analysed in this study only represents half of the completed trials by any given subject, as the original study also included lists that were to be forgotten as part of the original paradigm. Trials in these conditions are not further analysed in the context of this study.*

2) **As stated in the original manuscript, "there was no systematic temporal coupling between the delivery of the TMS pulses and the List 2 items." Was the presence of TMS artifact therefore even over the course of the epoch? This seems to be the assumption.**

TMS was administered at 1 Hz, meaning that in every epoch the distance between the pulses was identical and therefore the stimulation was even across trials. The jittered ISI ensured that no two trials would be exactly the same with regards to the timing of stimulation. As a sanity check we plotted the timings per stimulation condition (see the added supplementary material S3). Due to the fact that the ISI was jittered in discrete 0.25 second interval bins (=4Hz), there is a slight 4 Hz presence in the pulse timings. This increased the likelihood that stimulus presentation in the following trial occurred close to a time equal to a multiple of 0.25 seconds, corresponding to a 4 Hz bias due to the continuous 1 Hz stimulation in the background. However, this cannot be an explanation for the observed memory effects since this bias does not differ between the stimulation conditions. We confirmed this by performing a Kolmogorov Smirnoff test comparing the TMS pulse distributions between DLPFC and Vertex conditions. This test confirmed that the distributions of pulses are highly similar across conditions *(k-s statistic: 0.0295; p = 0.6709).* We made sure to add this analysis in the supplements as S3.

*S3: A) Raincloud plot of time difference between the first occurrence of a TMS pulse post word presentation for every trial (N=2400; 1200 per condition). Coloured area within the box-plots indicate the standard error, while the circles depict individual data points for each participant respectively. A slight 4 Hz bias in timing is visible in both conditions based on how the ISI was implemented. With a perfectly random ISI a uniform distribution would be expected. However, a two-sample Kolmogorov-Smirnov test confirmed that these two distributions do not statistically differ from each other* *(k-s statistic: 0.0295; p = 0.6709) .*

We also updated the section that the reviewer originally flagged, to reflect results of the aforementioned analysis.

*(line 420) The rTMS pulses and stimulus presentation were not synchronized by the experiment. Due to the nature the ISI being randomly chosen as a multiple of 0.25 seconds, there appeared to be a weak 4 Hz rhythm present (see supplementary figure S3). However, this bias did not systematically differ between stimulation conditions and therefore cannot explain the observed behavioral effects.*

3) **More detail on how "data around the rTMS artifacts [was] removed" (ln416) would be helpful. E.g., how did the authors remove these high-amplitude signals? Were they replaced with noise/zeros? How much time (in ms) does "around" mean in the above sentence?**

We are happy to provide more details on this important preprocessing step. Our analysis pipeline followed the procedure described in Herring et al. (2015, J Neurosci <https://www.jneurosci.org/content/35/43/14435.short>; for a tutorial see <https://www.fieldtriptoolbox.org/tutorial/tms-eeg/>) which employs a method using a combination of manual inspection and ICA. To clarify this further we added some of the requested details in the method section. The typical duration removed would be around ~10 ms, but that duration varied on a case-by-case basis due to slight idiosyncratic differences in the duration of the artifact. The reason it was not specified in the text, is that this part requires visual inspection and therefore is difficult to parameterize. The ICA itself separates the artifact from the rest of the signal, so this part does leave the data mostly intact. The subsequently removed data is then cubically interpolated. To provide the reader with a more detailed account we adapted the relevant methods section:

*(line 441) Preprocessing of rTMS-EEG data followed the guidelines and procedure outlined by Herring et al. [61]* *and described on the Fieldtrip tutorial website (*<https://www.fieldtriptoolbox.org/tutorial/tms-eeg/>*). EEG data were first cut into segments of -0.9s to 0.9s around the rTMS pulses. Data were visually inspected and data around the rTMS artifacts resulting from ringing and recharging of the stimulator were removed from further analysis**, as these can impact the performance of the subsequent pre-processing steps. The epoched data were subsequently subjected to an independent component analysis (runICA). This allowed the removal of rTMS related artefacts, eye-blink, eye movement and other remaining artefacts. Any missing data was interpolated with a cubic interpolation algorithm to avoid artificially induced artefacts in the data.*

4) **ln169: "Figure 5" should be "Figure 4".**

We thank the Reviewer for being so observant and have corrected this error.

5) **ln274, 287; The mechanistic inference between spectral tilt and parietal inhibition is ambiguous; the authors could clarify that the spectral slope is assumed to reflect the ratio between excitation and inhibition at the synaptic level with more negative slopes reflecting enhanced inhibition (Miller, PLoS Comp Biol, 2009).**

We made sure to make this statement more explicit in the text.

*(line 283) Previous research has suggested that the aperiodic component in electrophysiological signal may be the result of a neural ratio of excitation and inhibition in a local population of neurons [39]. Within this framework, the observed rotation would be associated with increased inhibition [40]. This would imply that the frontal stimulation has an inhibitory effect over the parietal cortex.*

**Academic Editor comments**

1) **General overview: The experimental design and overall protocol of the study is very difficult to grasp without going back and forth between introduction, results and methods sections. To aid the reader, I would suggest to give more information already earlier in the manuscript, and present the design and overall protocol in a more detailed schematic than currently done with Figure 1. For example, include information about the number of runs in the results already, as the reader in the current presentation might get the impression that the experiments included only 2x10 words in total.**

We agree with the editor that a more detailed depiction of the experimental procedure would aid the readers understanding. A similar point was also made by reviewer #1. To address this issue, we revised figure 1 which now should provide all the relevant information without needing the reader to go back to the method section to understand the critical aspects of the paradigm. In addition, we added some more information in the methods section. We hope that these changes make the study clearer to a naïve reader.

*Fig 1. Experimental design. Arrows on brain model indicate stimulation site (DLPFC=purple, vertex=orange). Participants were asked to study two lists of 10 words over 12 runs. During encoding of list 2, 45 pulses of 1Hz rTMS were applied to the left DLPFC (MNI coordinates: -45, 6, 39) or vertex. Memory performance was assessed as percentage of correctly recalled words per list.*

2) **Timing of effects: I had some problems grasping the exact timing of the EEG analyses, both regarding methods and interpretation. In particular, to which time windows were the statistical analyses restricted - just the 1s post-stimulus (as indicated on p.23), or also before (as indicated in figure 3)? In the discussion, the authors interpret EEG differences after stimulus, however not those preceding the stimulus, which however seem in stronger need for an explanation. In the methods description, the authors report that there was no relationship between the timing of the rTMS pulses and the stimulus presentation, however do they have any timing information available, e.g. pulse triggers, which might help to shed some light on the timing of effects?**

The initially reported cluster-based permutation test was only performed on the 1s post-stimulus time period for the contrast DLPFC vs Vertex. A separate analysis was performed to test for a difference between L2 – L1 between the two stimulation conditions (DLPFC vs Vertex). This second analysis (as depicted in Figure 3D) was performed with binned time-points across a longer time-window including pre and post stimulus. This confusion likely arose, due to it only being described in the results section but not the method section. We made sure to rectify this issue and describe this second analysis in the method section as well.

*(line 479) To ensure that any observed effects were specific to stimulation trials, an additional analysis was performed comparing the List 1 and List 2 trials for the DLPFC and vertex groups respectively in a time-window from -1 s to 1.95 s relative to stimulus onset. This time-window was split into six non-overlapping time bins. The data was then analysed using a TIME (time bins) x LIST (List 1 vs List 2) x GROUP (DLPFC vs vertex) ANOVA accompanied by post-hoc independent samples t-tests.*

The issue of rTMS timing relative to stimulus presentation was also brought up by reviewer #2. Since the ISI was jittered in discrete 0.25 second interval bins (=4Hz), there is a slight 4 Hz presence in the pulse timings. This increased the likelihood that stimulus presentation in the following trial occurred close to a time equal to a multiple of 0.25, which corresponds to 4 Hz, due to the continuous 1 Hz stimulation in the background. However, this cannot be an explanation for the observed memory effects since this bias does not differ between the stimulation conditions. We confirmed this by performing a Kolmogorov Smirnoff test comparing the TMS pulse distributions between DLPFC and Vertex conditions. This test confirmed that the distributions of pulses are highly similar across conditions *(k-s statistic: 0.0295; p = 0.6709) .* We made sure to add this analysis in the supplements as S3.

*S3: A) Raincloud plot of time difference between the first occurrence of a TMS pulse post word presentation for every trial (N=2400; 1200 per condition). Coloured area within the box-plots indicate the standard error, while the circles depict individual data points for each participant respectively. A slight 4 Hz bias in timing is visible in both conditions based on how the ISI was implemented. With a perfectly random ISI a uniform distribution would be expected. However, a two-sample Kolmogorov-Smirnov test confirmed that these two distributions do not statistically differ from each other (k-s statistic: 0.0295; p = 0.6709).*

We also updated the section the section in the methods that originally claimed that there was no relationship between stimulus timing and rTMS:

*(line 420) The rTMS pulses and stimulus presentation were not synchronized by the experiment. Due to the nature the ISI being randomly chosen as a multiple of 0.25 seconds, there appeared to be a weak 4 Hz rhythm present (see supplementary material S3). However, this bias did not systematically differ between stimulation conditions and therefore cannot explain the observed behavioral effects.*

3) **General interpretation of effects: Given that brains tend to be optimized during evolution, enhancement effects through brain stimulation are more interesting, but also more difficult to explain than impairing effects. If I understood it correctly, the authors hypothesize that their rTMS protocol led to disinhibiting effects. However, again from an evolutionary point of view, decreasing an existing inhibition should be even easier to evolve than a novel positively exciting effect - which makes the results somewhat puzzling. I would find it helpful if the authors could elaborate a bit more on two points in this regard:**
   a. **in how far can the authors exclude the possibility that vertex stimulation decreases rather than DLPFC stimulation increases memory performance?**

This is a fair point that we initially not had considered, because it seems unlikely given the existing literature. For instance, a study using 1 Hz TMS has illustrated that this type of stimulation over the vertex does not affect the wider brain, other than minor local activity changes (Jung, Bungert, Bowtell, & Jackson, 2016, *Brain stimulation,* [*https://doi.org/10.1016/j.brs.2015.09.008*](https://doi.org/10.1016/j.brs.2015.09.008)), which led Jung et al to conclude that the vertex is a good candidate for control conditions in TMS experiments. This is in stark contrast to the DLPFC which has been shown to be critically important for episodic memory encoding (Blumenfeld, & Ranganath, 2007 *The Neuroscientist,* [*https://doi.org/10.1177/1073858407299290*](https://doi.org/10.1177%2F1073858407299290)). Therefore, we would argue that while we cannot exclude the possibility that vertex stimulation decreases led to the observed effects, this seems very unlikely. However, based on the current experiment, this difference cannot be completely disentangled. Therefore, we made sure to mention this possibility in the in the discussion.

*(line 358) Another caveat inherent to the experiment is that due to the lack of a no stimulation condition for list 2, we are unable to completely exclude the possibility that Vertex stimulation reduces memory performance instead of DLPFC enhancing memory performance.* *A previous study using 1 Hz TMS and measuring fMRI BOLD signal concurrently showed that vertex stimulation does not affect the wider brain other than minor local changes, suggesting that vertex stimulation is a good control site* [52]*.*

Additionally, we added a clarifying statement in the rTMS section of the methods, further qualifying the choice of the vertex as a control region:

*(line 419) The vertex was chosen as a control site, as it has been shown to not have any wide-ranging network effects for 1 Hz stimulation [52].*

b. **Which memory-relevant processes specifically might be (dis-)inhibited, and in how far would this lead to e.g. more efficient neural coding?**

The DLPFC is implicated in a multitude of higher-order functions, such as attention, cognitive planning, inhibition (in the context of lying or action-inhibition), decision making, and working memory among many others. With an area involved in so many non-specific higher-order functions that each could contribute to memory in one way or another it is almost arbitrary to assign an interpretation. Given the data at hand we can only speculate about the cognitive processes based on the brain regions highlighted in our data. However, reasoning backward from neuroimaging data in such a way is not deductively valid (Poldrack, 2006, *Trends in cognitive sciences, https://pubmed.ncbi.nlm.nih.gov/16406760/*). Therefore, we prefer to refrain from making any further conclusions of this sort. The disinhibition hypothesis put forward in the manuscript is one of multiple suggested possible mechanisms. Further research will be required to pinpoint what the exact mechanism is at play here, or whether it is disinhibition at all. It is possible that the observed effects are the result of network-determined effects, which are also discussed as viable alternatives that cannot be discounted yet, among others.

4) **Blinding: The authors present the second study - already in the abstract - as conducted double blind. However, they use a rather non-standard definition of double blinding, namely simply being blind towards the specific hypotheses. According to this definition, likely the majority of studies would have to be considered at least single-blind, and many studies where data acquisition is done by research assistants with little involvement in the design and interpretation would have to be considered double blind this way. Under this definition, even participants of a pharmacological study who receive open label medication would have to be considered blinded as long as they aren't told explicitly which effects have been hypothesized for verum vs. placebo - which certainly would be anything but double blind according to the common use of the term: namely that participants and researchers can't tell which condition they are in during the experiment. I would thus recommend not to Speak of 'double blind' altogether, and/or at least make it very clear throughout the abstract and manuscript how this term is used here.**

When we included the term double-blind in the manuscript we were referring to the fact that both the subject and the experimenter were blind to the manipulation. The subjects are stimulated in both conditions, and while they can feel the stimulation on different parts of their head, there was no reason to believe that they were aware of the hypotheses. This was further supported by the fact that the experimenter was also blind to the hypotheses and therefor could not accidentally bias the performance of the participants in a specific direction. After further consideration, however, we realise that the use of the term double-blind in the abstract could be misleading and does not conform to its usual definition. We therefore removed any reference to the study design being double-blinded.

5) **In Figure 4A, the y axis lacks a label.**

We had originally not included any ticks on the Y axis, as these seemed arbitrary to us because that part of the figure is a schematic representation. However, we understand that a reader might be confused by the missing ticks and have added them accordingly.

 ***Fig 4. FOOOF analysis shows beta-effects are non-oscillatory in nature.*** *A) Schematic representation of the different components in a given power-spectrum. The black line represents a typical power-spectrum that is to be separated. The blue line is the corresponding log function following removal of the periodic peaks, thereby representing aperiodic properties of the signal. B) Power spectra separated by each condition. Shaded area indicates standard error. C)-D) Line plots of the mean aperiodic component before and after item presentation for the DLPFC and vertex condition respectively. The right axis relates to the plotted post-pre difference (dotted line).*
