# Supplementary material for: Stimulation of the left dorsolateral prefrontal cortex with slow rTMS enhances verbal memory formation
Source: PLoS Biol. 2021 Sep 28;19(9):e3001363. doi: 10.1371/journal.pbio.3001363 (PMC8478201; doi:10.1371/journal.pbio.3001363)
Supplement: Attachment — Submitted filename: Response to Reviewers - Revision 2.docx [file pbio.3001363.s008.docx]

**Response to Reviewers:**

We are very happy that we were able to address the previous points of contention to the reviewers satisfaction and are happy to address these minor outstanding issues brought up by reviewer 1. Just as in the previous response, we provide the original points of the reviewers in Bold, our response to the comment in plain text, and the changed section in the manuscript in Italics. Within the manuscript, changes are indicated with comments and tracked changes (line references refer to the untracked version).

**Reviewer #1: The authors have done an impressive job responding to reviewer concerns and have greatly improved the manuscript. I only have two minor comments that are non-essential in nature:**

**1. The additional detail regarding the temporal clustering analysis is helpful for the reader. However, it was still unclear to me how temporal distances were computed. Specifically, it is unclear whether 'position during encoding' and expected distance resets at the beginning of each list (i.e., does the first item in List 2 start at position 1 or position 11). This information is necessary to interpret the temporal clustering results. This detail is not provided in the referenced paper, which does not have multiple lists per recall period. Thus, it is unclear whether this measure provides information regarding clustering between as opposed to within the two lists.**

The reviewer brings up an important detail relating to the temporal clustering values that was left implicit. Since recall of both lists occurred at the same time, List 1 and List 2 items had to be coded uniquely if we want to compare the lists across the two conditions to each other. Specifically, both lists were treated as one large list of 20 items with List 1 items being coded as positions 1-10, and List 2 items being coded as positions 11-20. Therefore, with the 2 x 2 ANOVA we are investigating the temporal clustering within and between list categories. We made sure to explicitly state this in the manuscript as described in the following section.

*(line 552) This yielded a temporal clustering value for each list and condition per participant (see supplementary material S6 for values per condition). The items in list 1 were coded with the numbers 1-10, while items belonging to the second list were coded with numbers 11-20. These were then directly compared to each other using a 2x2 repeated measure ANOVA (List x Stimulation Condition).*

**2. In the discussion, the authors mention (line 345) that the memory effect (of stimulation) was specific for list 2 words. However, the behavioral analysis showed a positive effect of stimulation on items at the end of list 1 in experiment 1. Although the experimental design makes it difficult to interpret this effect (because both lists were recalled at the same time, see my previous major concern 1), this point seems rather important in interpreting the effects of stimulation, and I would tone down this claim. However, these are exciting results, and I am curious to know how and whether they replicate outside of the directed forgetting paradigm.**

We can see where the reviewer is coming from regarding this claim on list 2 specificity in experiment 1. We had originally made this statement, based on the significant interaction effect between word list and stimulation condition. However, as the reviewer points out, the statement leaves out some finer details regarding individual positions. We had not worried about the intensity of the claim, as we did not find this interaction effect in experiment 2. Therefore, as per the reviewers request we have toned down the claim in line 345 in the following way:

*(line 344) Considering the first experiment, the memory effect was not only specific to the DLPFC stimulation condition compared to the vertex condition, but also* *significantly stronger for list 2 words (i.e. those words that were presented during rTMS) as indicated by the interaction between words list and stimulation condition.*
